# Fatores Associados aos Custos do Tratamento no Primeiro Ano após Implantes Iniciais ou Trocas de Geradores de Pulsos de Marca-passos Cardíacos

**DOI:** 10.36660/abc.20230386

**Published:** 2024-04-11

**Authors:** Lucas Bassoli de Oliveira Alves, Katia Regina Silva, Jacson Venancio Barros, Fernando Antonio Basile Colugnati, Martino Martinelli, Roberto Costa

**Affiliations:** 1 Hospital das Clínicas Faculdade de Medicina Universidade de São Paulo São Paulo SP Brasil Instituto do Coração do Hospital das Clínicas da Faculdade de Medicina da Universidade de São Paulo, São Paulo, SP – Brasil; 2 Faculdade de Medicina Universidade de São Paulo São Paulo SP Brasil Faculdade de Medicina da Universidade de São Paulo (FMUSP), São Paulo, SP – Brasil; 3 Universidade Federal de Juiz de Fora Juiz de Fora MG Brasil Universidade Federal de Juiz de Fora, Juiz de Fora, MG – Brasil

**Keywords:** Marca-passo Artificial, Complicações Pós-operatórias, Readmissão Hospitalar, Custos Hospitalares, Avaliação em Saúde

## Abstract

**Fundamento:**

O uso de marca-passos cardíacos artificiais tem crescido constantemente, acompanhando o envelhecimento populacional.

**Objetivos:**

Determinar as taxas de readmissões hospitalares e complicações após implante de marca-passo ou troca de gerador de pulsos e avaliar o impacto desses eventos nos custos anuais do tratamento sob a perspectiva do Sistema Único de Saúde (SUS).

**Métodos:**

Registro prospectivo, com dados derivados da prática clínica assistencial, coletados na hospitalização índice e durante os primeiros 12 meses após o procedimento cirúrgico. O custo da hospitalização índice, do procedimento e do seguimento clínico foram estimados de acordo com os valores reembolsados pelo SUS e analisados ao nível do paciente. Modelos lineares generalizados foram utilizados para estudar fatores associados ao custo total anual do tratamento, adotando-se um nível de significância de 5%.

**Resultados:**

No total, 1.223 pacientes consecutivos foram submetidos a implante inicial (n= 634) ou troca do gerador de pulsos (n= 589). Foram observados 70 episódios de complicação em 63 pacientes (5,1%). A incidência de readmissões hospitalares em um ano foi de 16,4% (IC 95% 13,7% - 19,6%) após implantes iniciais e 10,6% (IC 95% 8,3% - 13,4%) após trocas de geradores. Doença renal crônica, histórico de acidente vascular encefálico, tempo de permanência hospitalar, necessidade de cuidados intensivos pós-operatórios, complicações e readmissões hospitalares mostraram um impacto significativo sobre o custo anual total do tratamento.

**Conclusões:**

Os resultados confirmam a influência da idade, comorbidades, complicações pós-operatórias e readmissões hospitalares como fatores associados ao incremento do custo total anual do tratamento de pacientes com marca-passo.

## Graphical abstract

**Figura Central:**
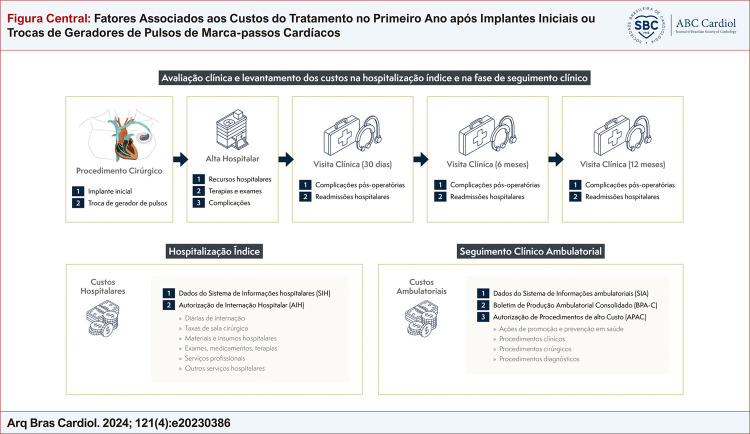
: Fatores Associados aos Custos do Tratamento no Primeiro Ano após Implantes Iniciais ou Trocas de Geradores de Pulsos de Marca-passos Cardíacos

## Introdução

O uso de marca-passos cardíacos artificiais tem crescido constantemente, acompanhando o envelhecimento populacional. Este tipo de tratamento tem sido realizado com baixas taxas de complicações perioperatórias e com efeito comprovado para o aumento da sobrevivência e da remissão dos sintomas.^1-3^ Estudos embasados em análise de dados de sistemas administrativos, entretanto, têm demonstrado aumento progressivo das taxas de complicações pós-operatórias e de readmissões hospitalares, que têm sido explicados, principalmente, pela fragilidade e pelas comorbidades desta população.^4-8^

Complicações pós-operatórias e readmissões hospitalares são importantes indicadores da qualidade assistencial e, por isso, têm sido cada vez mais estudados.^9-15^ Além dos impactos negativos que causam à saúde dos pacientes, estes eventos são uma das principais fontes de custos inesperados para o sistema de saúde, implicando na ineficiência operacional dos leitos hospitalares e na redução da capacidade de atendimento dos serviços especializados.^16-19^

A principal lacuna dessa área do conhecimento, entretanto, diz respeito à falta de dados resultantes da prática clínica real, visto que, a maioria dos estudos de avaliação econômica em estimulação cardíaca artificial têm sido embasados em métodos de modelagem estatística a partir de dados de estudos clínicos controlados.^20,21^Embora esses estudos tenham grande valor científico, a extrapolação dos resultados para o nosso contexto nem sempre é possível, especialmente, por envolverem amostras populacionais homogêneas, com condições clínicas restritas e tratamentos controlados, dificilmente reprodutíveis no nosso modelo de assistência.

A finalidade do presente estudo foi determinar as taxas de readmissões hospitalares e complicações após implante de marca-passo ou troca de gerador de pulsos e avaliar o impacto desses eventos nos custos anuais do tratamento de pacientes com marca-passo sob a perspectiva do Sistema Único de Saúde (SUS).

## Métodos

### Desenho e local do estudo

Trata-se de um registro prospectivo com dados derivados da prática clínica assistencial realizado em um hospital cardiológico terciário localizado na cidade de São Paulo.

Os dados foram coletados em quatro momentos distintos: na hospitalização índice relacionada ao procedimento cirúrgico e aos 30 dias, 6 meses e 12 meses após a alta hospitalar (Figura Central).

### População do estudo

Foram incluídos, consecutivamente, todos os pacientes adultos submetidos a implante inicial de marca-passo ou troca de gerador de pulsos, independentemente da indicação clínica e da técnica cirúrgica utilizada. Não foram incluídos pacientes que tiveram seu tratamento custeado por fontes privadas de financiamento ou que necessitavam de procedimentos associados à troca do gerador de pulsos, como o implante ou a remoção de cabos-eletrodos.

### Estimativa dos custos do tratamento

O método escolhido para a estimativa de custos foi o macrocusteio, considerando-se apenas os custos diretos do tratamento reembolsados pelo SUS para nossa instituição.^22^

Para o levantamento dos custos foi construído um repositório com os dados individualizados de todos os pacientes incluídos no estudo provenientes das bases de faturamento do SUS. A Autorização de Internação Hospitalar (AIH), foi a unidade básica de apuração dos custos relacionados aos episódios de internação hospitalar, enquanto a Autorização de Procedimento de Alto Custo (APAC) e o Boletim de Produção Ambulatorial (BPA), foram os sistemas utilizados para o levantamento dos custos em nível ambulatorial.

Para a estimativa do custo total da hospitalização índice foram considerados os serviços hospitalares (diárias de enfermaria ou unidade de intensiva, materiais hospitalares, exames de imagem e laboratoriais, medicamentos, terapias concomitantes) e os honorários profissionais referentes aos serviços médicos. Esses custos foram calculados com base em valores fixos que foram reembolsados pelo SUS a partir da apresentação da AIH após a alta do paciente.

O custo do procedimento, incluindo o dispositivo cardíaco, os cabos-eletrodos e outros insumos, foi calculado de acordo com a Tabela de Procedimentos, Medicamentos, Órteses, Próteses e Materiais Especiais (OPM) do SUS.^23^

Os custos relacionados à fase de seguimento clínico incluíram todos os atendimentos e procedimentos ambulatoriais, exames diagnósticos, exames laboratoriais, assim como, as readmissões hospitalares e intervenções cirúrgicas.

### Desfechos do estudo

Os desfechos estudados foram as readmissões hospitalares, as complicações pós-operatórias e os custos do tratamento.

Foram considerados todos os episódios de readmissões, por qualquer motivo, ocorridos no primeiro ano após a alta da hospitalização índice. A incidência de readmissões foi medida em dois momentos: em até 30 dias após a alta hospitalar (readmissão precoce) e no final do seguimento de um ano (readmissão tardia).

As complicações pós-operatórias incluíram: pneumotórax, hemotórax, perfurações ou lesões de estruturas cardíacas, problemas na loja do gerador de pulsos com necessidade de intervenção, infecção local ou sistêmica relacionada ao dispositivo, endocardite, trombose venosa do membro ipsilateral ao dispositivo e disfunção de cabos-eletrodos.

Os custos do tratamento foram representados pela soma dos valores, em reais (R$), reembolsados pelo SUS para as despesas relacionadas à hospitalização índice, ao procedimento cirúrgico, ao seguimento clínico dos pacientes durante os primeiros 12 meses de tratamento e às eventuais readmissões hospitalares ocorridas no período do estudo.

### Coleta e gerenciamento dos dados

Os dados do estudo foram coletados em formulários eletrônicos desenvolvidos no software REDCap (*Research Electronic Data Capture*)^24^ hospedado em nossa Instituição. Ao longo de todo o estudo, foram utilizadas funcionalidades específicas do REDCap para o monitoramento da qualidade dos dados.

### Variáveis estudadas e análise estatística

Para a análise dos resultados foram consideradas as variáveis demográficas (idade, sexo, escolaridade, estado empregatício), pré-operatórias (tipo de hospitalização, doença cardíaca estrutural, medicamentos de uso regular, comorbidades, fração de ejeção do ventrículo esquerdo obtida por ecocardiografia transtorácica bidimensional), cirúrgicas (tipo de procedimento, indicação do marca-passo, tipo de marca-passo) e as características da admissão hospitalar índice.

As variáveis contínuas foram descritas em mediana e intervalo interquartil (IQR) e as variáveis categóricas foram descritas em frequências absolutas e relativas. Os testes qui-quadrado de Pearson, exato de Fisher e Mann-Whitney foram utilizados para a comparação das características basais dos dois grupos estudados.

A incidência de readmissão hospitalar foi descrita em probabilidade percentual e intervalos de confiança (IC) de 95%, segundo o método de Kaplan-Meier. O teste de log-rank foi utilizado para comparação das estimativas entre os dois grupos estudados.

Empregou-se o método de regressão de riscos proporcionais de Cox para estudar fatores associados às readmissões hospitalares. As variáveis com valores de p < 0,10 na análise univariada foram selecionadas para o modelo multivariado final. Os resultados do modelo final foram apresentados em *Hazard Ratio* (HR) e seus respectivos IC 95%. O ajuste do modelo final foi avaliado pelo cálculo do índice de concordância do modelo (índice-C). Valores de índice-C iguais ou superiores a 0,70 foram considerados satisfatórios.

O custo do tratamento foi descrito segundo valores médios não ajustados (amostrais) e ajustados (preditos) seguidos do IC 95%, obtidos pela técnica de *bootstrap* para 5.000 reamostragens não-paramétricas. Para identificar fatores associados ao custo total anual do tratamento, foram utilizados modelos lineares generalizados (*GLM*) simples e multivariados, utilizando a função *log-link* e a distribuição *Gamma* para modelagem do custo anual total do tratamento. Covariáveis com p-valor inferior a 0,10 (na análise univariada) foram incluídas no modelo multivariado final.

A análise estatística foi realizada no software R Studio, adotando-se um nível de significância de 5% para todos os testes de hipóteses realizados.

### Aspectos éticos

O estudo, realizado no período de janeiro de 2014 a dezembro de 2018, foi aprovado pelo Comitê de Ética em Pesquisa da Instituição. Por se tratar de um estudo observacional, com dados derivados da prática assistencial e obtidos diretamente dos sistemas hospitalares (prontuário eletrônico do paciente e sistemas de dados administrativos), o estudo isentou-se da necessidade de assinatura do Termo de Consentimento Livre e Esclarecido.

## Resultados

### Composição da amostra

Durante o período do estudo, 1.418 pacientes foram submetidos a procedimentos cirúrgicos para o implante inicial ou a troca do gerador de pulsos de marca-passos cardíacos convencionais. Destes, 44 pacientes possuíam idade inferior a 18 anos e outros 151 pacientes tiveram seu tratamento custeado por fontes privadas de financiamento, sendo considerados não elegíveis para o estudo. A amostra final foi composta por 1.223 pacientes, sendo 634 submetidos ao implante inicial e 589 a troca do gerador do marca-passo.

### Características basais

A amostra apresentou maior frequência de mulheres e idade mediana de 73 anos (Q1-Q2: 63–81 anos). A proporção de mulheres e a idade mediana foram maiores entre os pacientes do grupo troca de gerador. A frequência de comorbidades foi maior no grupo de implante de marca-passo, conforme descrito na Tabela 1.

**Tabela 1:** – Características basais dos pacientes submetidos a implante inicial ou troca de gerador de pulsos de marca-passos

Características basais	Amostra total n= 1.223	Implante inicial n= 634	Troca de gerador n= 589	p
Sexo feminino, n (%)	687 (56,2)	334 (52,7)	353 (59,9)	0,010
**Idade (anos) mediana (IQR), n (%)**	73,0 (63 – 81)	72,0 (64 – 80)	74,0 (63 – 82)	< 0,001
< 60	230 (18,8)	112 (17,7)	118 (20,0)	0,030
60 – 69	255 (20,9)	149 (23,5)	106 (18,0)	
70 – 79	392 (32,1)	213 (33,6)	179 (30,4)	
80 – 89	296 (24,2)	137 (21,6)	1859 (27,0)	
≥ 90	50 (4,1)	23 (3,6)	27 (4,6)	
Escolaridade elementar, n (%)	864 (70,6)	423 (66,7)	441 (74,8)	0,621
Aposentados, n (%)	544 (44,5)	267 (42,1)	277 (47,0)	0,087
**Comorbidades, n (%)**				
Hipertensão	872 (71,3)	460 (72,6)	412 (69,9)	0,314
Diabetes mellitus	306 (25,0)	180 (28,4)	126 (21,4)	0,005
Doença valvar	241 (19,7)	141 (22,2)	100 (17,0)	0,021
Fibrilação atrial	259 (21,2)	125 (19,7)	134 (22,7)	0,208
Doença arterial coronariana	173 (14,1)	112 (17,7)	61 (10,4)	< 0,001
Doença renal crônica	110 (9,0)	72 (11,4)	38 (6,4)	0,003
Acidente vascular encefálico	91 (7,4)	58 (9,1)	33 (5,6)	0,018
**Doença cardíaca estrutural, n (%)**				
Não-isquêmica	181 (14,8)	93 (14,7)	88 (14,9)	0,852
Isquêmica	46 (3,7)	27 (4,2)	19 (3,2)	0,355
Chagásica	199 (16,3)	97 (15,3)	102 (17,3)	0,310
Fração de ejeção do VE < 40%, n (%)	72 (5,9)	38 (6,0)	34 (5,7)	0,467
**Indicação do marca-passo, n (%)**				
Doença do nó sinusal	123 (10,1)	61 (9,6)	62 (10,5)	0,102
Bloqueio atrioventricular avançado	1,028 (84,0)	527 (83,1)	501 (85,0)	
Outras indicações	72 (5,9)	46 (7,2)	26 (4,4)	
**Características do procedimento cirúrgico, n (%)**				
Marca-passo câmara dupla	1,059 (86,6)	549 (86,6)	510 (86,6)	0,998
Acesso transvenoso	1,209 (98,8)	624 (98,4)	585 (99,3)	0,962
Procedimento eletivo	217 (17,7)	57 (9,0)	160 (27,2)	< 0,001
**Hospitalização, n (%)**				
Hospitalização em regime de urgência	531 (43,4)	473 (74,6)	58 (9,8)	< 0,001
Cirurgia realizada no mesmo dia da admissão	217 (17,7)	57 (9,0)	160 (27,2)	< 0,001
Tempo de permanência hospitalar > 3 dias	473 (38,7)	409 (64,5)	64 (10,9)	< 0,001
Tempo de permanência pós-operatório > 1 dia	170 (13,9)	154 (24,3)	16 (2,7)	< 0,001
Diárias de UTI no pós-operatório	101 (8,3)	97 (15,3)	4 (0,7)	< 0,001
**Medicação de uso regular, n (%)**				
Agentes antiplaquetários	470 (38,4)	243 (38,3)	227 (38,5)	0,924
Anticoagulantes orais	139 (11,4)	62 (9,8)	77 (13,1)	0,084
IECA/BRA	837 (68,4)	394 (62,1)	443 (75,2)	< 0,001
Beta-bloqueadores	430 (35,1)	130 (20,5)	300 (50,9)	< 0,001
Diuréticos	609 (49,8)	316 (49,8)	293 (49,7)	0,848
Antiarrítmicos	94 (7,7)	43 (6,8)	51 (8,6)	0,243

BRA: bloqueador do receptor de angiotensina; IECA: inibidor da enzima conversora de angiotensina; IQR: intervalo interquartil; UTI: unidade de terapia intensiva; VE: ventrículo esquerdo.

Aproximadamente três em cada quatro implantes iniciais foram realizados em regime de urgência, enquanto uma pequena parcela dos procedimentos de troca de gerador ocorreu em caráter de urgência. A taxa de procedimento cirúrgico realizado no mesmo dia da admissão hospitalar foi aproximadamente significativamente maior no grupo de troca do gerador. Os dispositivos de câmara dupla implantados por acesso transvenoso foram os mais frequentes na amostra. A necessidade de cuidados intensivos e o tempo de permanência pós-operatória foram significativamente maiores no grupo de implantes iniciais (Tabela 1).

### Óbitos, complicações pós-operatórias e readmissões hospitalares

O tempo mediano de seguimento foi de 13,7 meses (Q1-Q2: 12,3-14,8 meses). Apenas um paciente foi perdido para seguimento.

Foram observados 109 óbitos durante o primeiro ano de seguimento, representando uma mortalidade cumulativa de 8,9% (IC 95% 7,4%-10,6%). As causas de óbitos estão descritas na Tabela 2.

**Tabela 2:** – Taxa de complicações pós-operatórias, readmissões hospitalares e óbitos após implante inicial ou troca de gerador de pulsos de marca-passos

Desfechos	Amostra total n= 1.223	Implante inicial n= 634	Troca de gerador n= 589
**Complicações pós-operatórias, n (%)**			
Pneumotórax	12 (1,0)	11 (1,7)	1 (0,2)
Tamponamento cardíaco	3 (0,2)	3 (0,5)	0 (-)
Deslocamento de cabo-eletrodo	11 (0,9)	10 (1,6)	1 (0,2)
Disfunção de cabo-eletrodo	8 (0,7)	1 (0,2)	7 (1,2)
Problemas na conexão entre o gerador e os cabos-eletrodos	1 (0,1)	1 (0,2)	0 (-)
Complicações de loja	20 (1,6)	8 (1,3)	12 (2,3)
Infecção do dispositivo	8 (0,7)	5 (0,8)	3 (0,5)
Trombose venosa profunda	4 (0,3)	3 (0,5)	1 (0,2)
Estimulação muscular	3 (0,2)	1 (0,7)	2 (0,3)
Qualquer complicação	63 (5,2)	38 (6,0)	25 (4,2)
**Readmissões hospitalares precoces, n (%)**			
Relacionada ao marca-passo	13 (1,1)	9 (1,4)	4 (0,7)
Insuficiência cardíaca	5 (0,4)	5 (0,8)	0 (-)
Outras causas cardiovasculares	1 (0,1)	0 (-)	1 (0,2)
Não cardiovascular	14 (1,1)	13 (2,2)	1 (0,2)
**Readmissões hospitalares tardias, n (%)**			
Relacionada ao marca-passo	17 (1,4)	5 (0,8)	12 (2,0)
Insuficiência cardíaca	20 (1,6)	14 (2,2)	6 (1,0)
Outras causas cardiovasculares	28 (2,3)	10 (1,6)	18 (3,0)
Não cardiovascular	64 (5,2)	45 (7,1)	19 (3,2)
**Óbitos, n (%)**			
Relacionado ao marca-passo	4 (0,3)	3 (0,5)	1 (0,2)
Insuficiência cardíaca	6 (0,5)	2 (0,3)	4 (0,7)
Outras causas cardiovasculares	29 (2,4)	14 (2,2)	15 (2,5)
Não cardiovascular	63 (5,1)	46 (7,2)	17 (2,9)
Causa indeterminada	7 (0,6)	5 (0,8)	2 (0,3)

Setenta episódios de complicações foram detectados em 63 pacientes. Dentre as complicações pós-operatórias, 27 ocorreram durante a hospitalização índice. Essas complicações aumentaram significativamente o tempo de permanência hospitalar e a necessidade de cuidados intensivos. A mediana do tempo de internação foi de 5,0 dias (Q1-Q2: 2,5-12) no grupo que apresentou complicações e de 1,0 dia (Q1-Q3: 0-1,0) no grupo que não apresentou complicações. Os tipos de complicações pós-operatórias estão descritos na Tabela 2.

**Figura 1:**
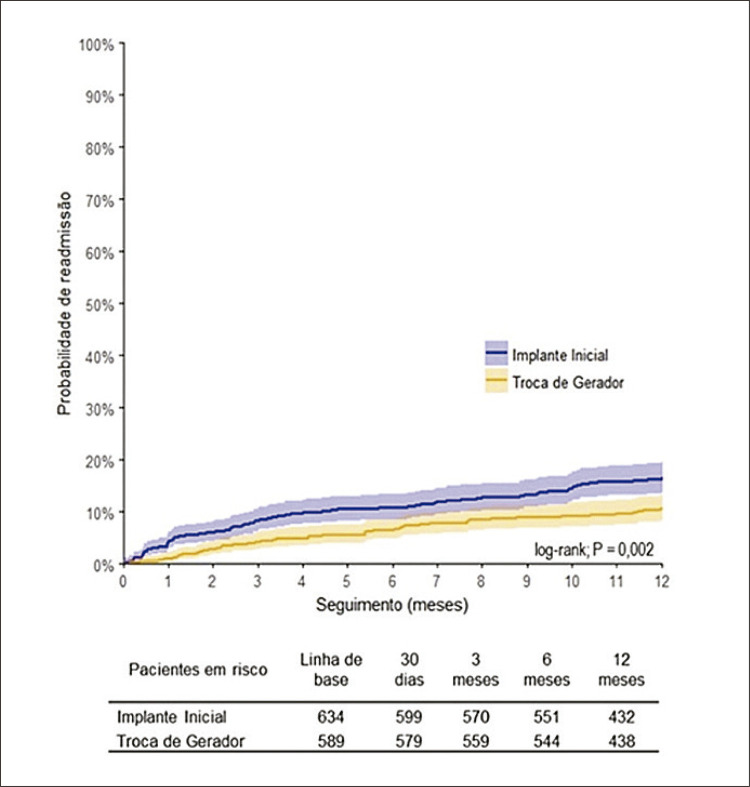
– Probabilidade de readmissão em 12 meses, segundo o tipo de procedimento cirúrgico realizado.

A incidência de readmissões em até 30 dias foi de 4,3% (IC 95%: 3,0%-6,2%) para o grupo implante inicial e de 1,0% (IC 95%: 0,5%-2,3%) para o grupo troca do gerador. As readmissões hospitalares ao final do primeiro ano de seguimento ocorreram em 16,4% (IC 95%: 13,7% - 19,6%) dos pacientes submetidos a implante inicial e em 10,6% (IC 95%: 8,3% - 13,4%), dos pacientes submetidos à troca do gerador (Figura 1). As causas de readmissões hospitalares estão descritas na Tabela 2 e os fatores independentes para a sua ocorrência estão descritos na Tabela 3.

**Tabela 3:** – Preditores de readmissão hospitalar após implante inicial ou troca de gerador de pulsos de marca-passos

Fatores de risco	Hazard Ratio (IC 95%)	p
**Implante de marca-passo^**1**^**		
Idade ≥ 90 anos	1,29 (0,59 – 2,82)	0,522
Diabetes mellitus	1,47 (0,94 – 2,29)	0,087
Doença renal crônica	2,02 (1,21 – 3,39)	0,007
Fibrilação atrial	1,72 (0,99 – 2,96)	0,051
Acidente vascular encefálico prévio	1,20 (0,64 – 2,21)	0,566
Doença cardíaca estrutural	1,56 (1,01 – 2,40)	0,043
Indicação do implante de marca-passo		
Bloqueio atrioventricular	referência	-
Doença do nó sinusal	0,75 (0,34 – 1,66)	0,482
Outras indicações	1,24 (0,60 – 2,54)	0,563
Uso regular de anticoagulantes orais	0,70 (0,36 – 1,38)	0,309
Marca-passo de câmara única	2,61 (1,55 – 4,41)	< 0,001
Tempo de permanência hospitalar no pós-operatório > 1 dia	1,07 (0,63 – 1,81)	0,799
Tempo de permanência hospitalar > 3 dias	0,90 (0,55 – 1,49)	0,692
Necessidade de UTI no pós-operatório	1,14 (0,66 – 1,97)	0,640
Complicações relacionadas ao marca-passo	5,94 (3,33 – 10,58)	< 0,001
**Troca de gerador de pulsos de marca-passo ^**2**^**		
Idade ≥ 80 anos	2,52 (1,48 – 4,29)	< 0,001
Sexo masculino	1,41 (0,82 – 2,42)	0,214
Doença renal crônica	2,17 (0,99 – 4,74)	0,052
Fibrilação atrial	1,18 (0,58 – 2,39)	0,640
Acidente vascular encefálico prévio	2,93 (1,36 – 6,28)	0,006
Uso regular de anticoagulantes orais	1,03 (0,45 – 2,37)	0,935
Tempo de permanência hospitalar > 3 dias	1,67 (0,78 – 3,56)	0,181
Complicações relacionadas ao marca-passo	25,65 (12,70 – 51,60)	< 0,001

^1^ n = 596; índice-C = 0,757; ^2^ n = 582; índice-C = 0,815.

### Custo do tratamento no primeiro ano após o procedimento

Para o tratamento dos pacientes incluídos no estudo, o SUS reembolsou a nossa instituição aproximadamente R$ 10,6 milhões. O dispositivo cardíaco, incluindo os cabos-eletrodos e o gerador de pulsos, foi o principal componente deste orçamento e representou mais de 70% do gasto anual total. A Tabela 4 apresenta uma descrição detalhada dos custos atribuídos ao tratamento dos pacientes dos grupos implante inicial e troca de gerador de pulsos.

**Tabela 4:** – Descrição dos gastos atribuídos à hospitalização índice, seguimento clínico e o montante total para o tratamento de pacientes com marca-passos

Componentes dos gastos	Média	IC 95%	Montante Total	Total
**Implante de marca-passo**				
**Total anual**	R$ 10.172	(9.770 – 10.620)	R$ 6.449.363	100%
**Implante do Dispositivo**				
**Total**	R$ 8.934	(8.702 – 9.205)	R$ 5.664.163	87,8%
Dispositivo (OPM)	R$ 7.162	(7.110 – 7.216)	R$ 4.540.877	70,4%
Internação Hospitalar	R$ 1.224	(1.155 – 1.307)	R$ 776.400	12,0%
Unidade de terapia intensiva	R$ 547	(366 – 750)	R$ 346.886	5,4%
**Seguimento ambulatorial**				
Consultas ou Procedimentos	R$ 743	(586 – 945)	R$ 471.157	7,3%
**Readmissões hospitalares**				
Total	R$ 495	(276 – 754)	R$ 314.043	4,9%
Dispositivo (OPM)	R$ 53	(19 – 97)	R$ 33.980	0,5%
Internação Hospitalar	R$ 442	(242 – 680)	R$ 280.063	4,4%
**Troca de gerador de pulsos**				
**Total anual**	R$ 7.092	(6.750 – 7.514)	R$ 4.177.440	100%
**Troca do gerador**				
Total	R$ 6.029	(5.994 – 6.068)	R$ 3.551.176	85,0%
Dispositivo (OPM)	R$ 5.125	(5.100 – 5.150)	R$ 3.018.836	72,3%
Internação Hospitalar	R$ 886	(871 – 904)	R$ 522.168	12,5%
Unidade de terapia intensiva	R$ 17	(0,86 – 42.3)	R$ 10.172	0,2%
**Seguimento ambulatorial**				
Consultas ou Procedimentos	R$ 510	(469 – 555)	R$ 300.350	7,2%
**Readmissões hospitalares**				
Total	R$ 553	(235 – 956)	R$ 325.914	7,8%
Dispositivo (OPM)	R$ 300	(77 – 593)	R$ 176.908	4,2%
Internação Hospitalar	R$ 253	(98 – 465)	R$ 149.007	3,6%

OPM: Órtese e prótese médica.

O modelo linear generalizado demonstrou que idade, doença renal crônica, acidente vascular cerebral prévio, tempo de permanência hospitalar maior que um dia, necessidade de cuidados intensivos pós-operatórios, complicações e readmissões hospitalares foram significativamente associados aos custos totais anuais do tratamento. A idade foi a única variável que se relacionou inversamente com o custo total do tratamento, independentemente do procedimento realizado (Tabela 5).

**Tabela 5:** – Modelo multivariado de fatores associados ao custo anual do tratamento de pacientes com marca-passo cardíaco

Fatores de risco	Exp B (95% IC)	p
**Idade grupos (anos)**		
< 60	Referência	--
60 – 69	0,97 (0,93 – 1,01)	0,149
70 – 79	0,96 (0,92 – 1,00)	0,035
80 – 89	0,92 (0,89 – 0,96)	< 0,001
≥ 90	0,87 (0,81 – 0,94)	< 0,001
Diabetes mellitus	1,03 (1,00 – 1,06)	0,086
Doença valvar	1,00 (0,96 – 1,03)	0,820
Fibrilação atrial	1,04 (1,00 – 1,08)	0,076
Doença arterial coronariana	1,01 (0,97 – 1,05)	0,579
Doença renal crônica	1,07 (1,02 – 1,12)	0,009
Acidente vascular encefálico prévio	1,08 (1,02 – 1,13)	0,005
Doença cardíaca estrutural	1,00 (0,97 – 1,03)	0,899
Uso regular de anticoagulantes orais	0,96 (0,91 – 1,01)	0,097
Hospitalização em regime de urgência	1,00 (0,96 – 1,04)	0,990
Procedimento realizado em caráter eletivo	0,99 (0,96 – 1,03)	0,787
Tempo de permanência hospitalar > 3 dias	1,00 (0,98 – 1,07)	0,298
Tempo de permanência hospitalar pós-operatório > 1 dia	1,00 (0,98 – 1,07)	0,298
Diárias de unidade de terapia intensiva no pós-operatório	1,39 (1,31 – 1,47)	< 0,001
Complicação relacionada ao marca-passo	1,17 (1,09 – 1,25)	< 0,001
Readmissão hospitalar	1,56 (1,48 – 1,66)	< 0,001

Exp B: exponencial do coeficiente beta.

**Figura 2:**
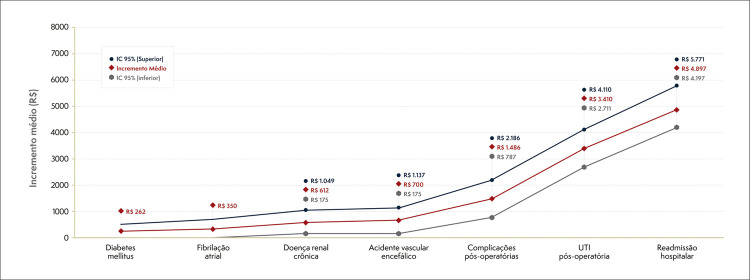
– Fatores associados ao incremento médio no custo total anual do tratamento de pacientes com marca-passo.

Readmissão hospitalar durante o seguimento clínico e a necessidade de cuidados intensivos pós-operatórios durante a hospitalização índice foram os principais fatores associado ao incremento do custo do tratamento, conforme detalhado na Figura 2.

## Discussão

Este estudo prospectivo com dados do mundo real mostrou que complicações pós-operatórias e readmissões hospitalares são frequentes após o implante inicial, assim como, após a troca do gerador de pulsos de marca-passos. Independentemente do motivo dessas readmissões, o impacto econômico sobre o custo total da saúde foi significativo para o sistema público de saúde.

Apesar de que pacientes submetidos a implante inicial ou à troca do gerador de pulsos façam parte da mesma população de pacientes, diferenças significativas foram observadas no perfil clínico e demográfico destes dois subgrupos, com maior proporção de mulheres, menor prevalência de comorbidades e maior frequência de uso de medicamentos cardiovasculares nos submetidos a troca de gerador de pulsos. Além disso, a maioria das substituições do gerador de pulsos foi realizada em internações eletivas, enquanto implantes iniciais foram realizados em sua maioria em regime de urgência. Essas diferenças influenciaram o resultado dos procedimentos, implicando em maiores tempo de permanência hospitalar, necessidade de cuidados em unidade de terapia intensiva e readmissões hospitalares nos pacientes submetidos a implantes iniciais. Embora os geradores de pulso atuais tenham expectativa de vida útil de aproximadamente 10 anos, a idade mediana dos pacientes no momento da substituição do gerador de pulsos excedeu a idade do grupo de implante inicial em apenas dois anos. Esse achado pode ser explicado pela alta taxa de pacientes que não chegam a ser submetidos à troca do gerador de pulsos pela idade avançada no momento do implante inicial.

As taxas de complicações pós-operatórias e de readmissões hospitalares precoces relacionadas ao procedimento cirúrgico ou ao dispositivo cardíaco encontradas neste estudo foram menores do que as reportadas nos estudos embasados em grandes bases de dados administrativos dos EUA, onde a taxa de readmissão hospitalar de 30 dias variou de 8,5% a 11,3%.^9,10^ No presente estudo, pneumotórax ou tamponamento cardíaco (2,2%) e complicações relacionadas ao cabo-eletrodo (1,9%) foram mais frequentes no grupo de implante inicial e suas taxas foram semelhantes às relatadas no estudo FOLLOWPACE (2,7% e 3,3%, respectivamente).^11^

Apesar da menor taxa total de readmissões no grupo troca de gerador, a frequência de readmissões relacionadas ao procedimento, foi maior neste subgrupo, principalmente após os primeiros 30 dias de seguimento. Essas readmissões foram relacionadas a complicações no alojamento do gerador de pulsos, disfunções de cabos-eletrodos e infecção relacionados ao dispositivo. De maneira semelhante ao reportado em outros estudos, essas complicações geralmente ocorreram tardiamente com necessidade de readmissão e revisão cirúrgica.^12-19^

A taxa de readmissão em um ano foi de 16,4% após implantes iniciais e de 10,6% no grupo troca de gerador. Idade, doença renal crônica, cardiopatia subjacente, marca-passo de câmara única e complicações pós-operatórias aumentaram significativamente o risco de readmissões, em concordância com outras publicações.^9,10,14^ Da mesma forma, doença renal crônica, acidente vascular cerebral prévio, complicações pós-operatórias e readmissões foram associados a maiores custos de saúde no primeiro ano, tanto após implante inicial quanto após substituição do gerador de pulsos. O monitoramento e o conhecimento das taxas de complicações pela equipe médica, o treinamento contínuo das equipes para a identificação de pacientes sob maior risco, além do seguimento multidisciplinar destes pacientes são medidas potencialmente acessíveis e que oferecem uma boa oportunidade de melhora nas taxas de complicações e readmissões após o procedimento.

Nossa análise revelou um impacto econômico significativo do custo do dispositivo no custo total do tratamento (cerca de 70%). Outras publicações já demonstraram a desproporção entre o custo do dispositivo cardíaco em relação a outras despesas, como as taxas de internação, exames laboratoriais, medicamentos e honorários profissionais.^18,19^

Este estudo apresenta algumas limitações que devem ser consideradas na interpretação dos resultados. Embora tenha sido incluída uma amostra muito representativa, esta análise reflete as práticas assistenciais de um centro público de cardiologia terciária, que também é um centro de treinamento para especialistas em estimulação cardíaca artificial. Os custos diretos foram calculados com base em valores fixos, ou pacotes que foram reembolsados pelo SUS, não tendo sido possível realizar a análise do microcusteio com dados detalhados sobre cada recurso utilizado e os custos unitários correspondentes a esses recursos. Finalmente, um acompanhamento a longo prazo dessa população é especialmente importante para fornecer evidências mais robustas sobre o impacto potencial de complicações tardias nos custos de saúde, que muitas vezes são subnotificados nesse cenário.

## Conclusões

O seguimento por até um ano de pacientes submetidos a implante inicial ou à troca de gerador de pulsos de marca-passo cardíaco artificial nos permitiu determinar a taxa de ocorrência de complicações pós-operatórias e de readmissões hospitalares, identificar fatores de risco para estes eventos e verificar que estes eventos implicaram aumento significativo do custo do tratamento. Os achados do presente estudo sugerem que a identificação dos pacientes que estão sob maior risco de manifestar estes eventos e a implementação de rotinas assistenciais específicas para seu seguimento ambulatorial pode resultar em redução significativa dos gastos com estes tipos de procedimento.

## References

[1] Westaway S, Nye E, Gallagher C, Tu SJ, Clarke N, Hanna-Rivero N (2021). Trends in the Use, Complications, and Costs of Permanent Pacemakers in Australia: a Nationwide Study from 2008 to 2017. Pacing Clin Electrophysiol.

[2] Vaidya VR, Asirvatham R, Kowlgi GN, Dai MY, Cochuyt JJ, Hodge DO (2022). Trends in Cardiovascular Implantable Electronic Device Insertion Between 1988 and 2018 in Olmsted County. JACC Clin Electrophysiol.

[3] Krishnaswami A, Liu TI, Harris J, Prentice HA, Paxton EW, Masoudi FA (2021). The Association of Multimorbidity to Mortality in Older Adults after Permanent Pacemaker Placement. Pacing Clin Electrophysiol.

[4] Kichloo A, Shaka H, Aljadah M, Amir R, Albosta M, Jamal S (2021). Predictors of Outcomes in Hospitalized Patients Undergoing Pacemaker Insertion: Analysis from the National Inpatient Database (2016-2017). Pacing Clin Electrophysiol.

[5] Cantillon DJ, Exner DV, Badie N, Davis K, Gu NY, Nabutovsky Y (2017). Complications and Health Care Costs Associated with Transvenous Cardiac Pacemakers in a Nationwide Assessment. JACC Clin Electrophysiol.

[6] Mohamed MO, Van Spall HGC, Morillo C, Wilton SB, Kontopantelis E, Rashid M (2022). The Impact of Charlson Comorbidity Index on De Novo Cardiac Implantable Electronic Device Procedural Outcomes in the United States. Mayo Clin Proc.

[7] Balla C, Malagu' M, Fabbian F, Guarino M, Zaraket F, Brieda A (2019). Prognosis after Pacemaker Implantation in Extreme Elderly. Eur J Intern Med.

[8] Antonelli D, Freedberg NA, Bushari LI, Feldman A, Turgeman Y (2015). Permanent Pacing in Nonagenarians Over 20-Year Period. Pacing Clin Electrophysiol.

[9] Patel B, Sablani N, Garg J, Chaudhary R, Shah M, Gupta R (2018). Thirty-Day Readmissions after Cardiac Implantable Electronic Devices in the United States: Insights from the Nationwide Readmissions Database. Heart Rhythm.

[10] Lemor A, Lee S, Dehkordi SHH, Mehta D (2017). Etiologies and Predictors for 30-day Readmission after Pacemaker Placement for Atrioventricular Block. A Nationwide Analysis. Eur Heart J.

[11] Udo EO, Zuithoff NP, van Hemel NM, Cock CC, Hendriks T, Doevendans PA (2012). Incidence and Predictors of Short- and Long-Term Complications in Pacemaker Therapy: the FOLLOWPACE Study. Heart Rhythm.

[12] Kirkfeldt RE, Johansen JB, Nohr EA, Jørgensen OD, Nielsen JC (2014). Complications after Cardiac Implantable Electronic Device Implantations: An Analysis of a Complete, Nationwide Cohort in Denmark. Eur Heart J.

[13] Poole JE, Gleva MJ, Mela T, Chung MK, Uslan DZ, Borge R (2010). Complication Rates Associated with Pacemaker or Implantable Cardioverter-Defibrillator Generator Replacements and Upgrade Procedures: Results from the REPLACE Registry. Circulation.

[14] Silva KR, Albertini CM, Crevelari ES, Carvalho EI, Fiorelli AI, Martinelli M (2016). Complications after Surgical Procedures in Patients with Cardiac Implantable Electronic Devices: Results of a Prospective Registry. Arq Bras Cardiol.

[15] Sohail MR, Eby EL, Ryan MP, Gunnarsson C, Wright LA, Greenspon AJ (2016). Incidence, Treatment Intensity, and Incremental Annual Expenditures for Patients Experiencing a Cardiac Implantable Electronic Device Infection: Evidence from a Large US Payer Database 1-Year Post Implantation. Circ Arrhythm Electrophysiol.

[16] Groeneveld PW, Dixit S (2017). Cardiac Pacing and Defibrillation Devices: Cost and Effectiveness. Annu Rev Med.

[17] Nichols CI, Vose JG, Mittal S (2016). Incidence and Costs Related to Lead Damage Occurring Within the First Year after a Cardiac Implantable Electronic Device Replacement Procedure. J Am Heart Assoc.

[18] Fanourgiakis J, Simantirakis E, Maniadakis N, Kourlaba G, Kanoupakis E, Chrysostomakis S (2013). Cost-of-Illness Study of Patients Subjected to Cardiac Rhythm Management Devices Implantation: Results from a Single Tertiary Centre. Europace.

[19] Oddershede L, Riahi S, Nielsen JC, Hjortshøj S, Andersen HR, Ehlers L (2014). Health Economic Evaluation of Single-Lead Atrial Pacing vs. Dual-Chamber Pacing in Sick Sinus Syndrome. Europace.

[20] Edwards SJ, Karner C, Trevor N, Wakefield V, Salih F (2015). Dual-Chamber Pacemakers for Treating Symptomatic Bradycardia due to Sick Sinus Syndrome without Atrioventricular Block: a Systematic Review and Economic Evaluation. Health Technol Assess.

[21] Deniz HB, Caro JJ, Ward A, Moller J, Malik F (2008). Economic and Health Consequences of Managing Bradycardia with Dual-Chamber Compared to Single-Chamber Ventricular Pacemakers in Italy. J Cardiovasc Med.

[22] Brasil, Ministério da Saúde, Secretaria de Ciência, Tecnologia e Insumos Estratégicos, Departamento de Ciência e Tecnologia (2014). Diretrizes Metodológicas - Estudos de Avaliação Econômica de Tecnologias em Saúde.

[23] Brasil, Ministério da Saúde (2023). SIGTAP - Sistema de Gerenciamento da Tabela de Procedimentos, Medicamentos e OPM do SUS.

[24] Harris PA, Taylor R, Thielke R, Payne J, Gonzalez N, Conde JG (2009). Research Electronic Data Capture (Redcap)-a Metadata-Driven Methodology and Workflow Process for Providing Translational Research Informatics Support. J Biomed Inform.

