# Supplementary material for: Fatores Associados aos Custos do Tratamento no Primeiro Ano após Implantes Iniciais ou Trocas de Geradores de Pulsos de Marca-passos Cardíacos
Source: Arq Bras Cardiol. 2024 Apr 11;121(4):e20230386. [Article in Portuguese] doi: 10.36660/abc.20230386 (PMC11098589; doi:10.36660/abc.20230386)
Supplement: Translation

# Translation: Factors Associated with the Treatment Costs within the First Year after Pacemaker Implantation or Pulse Generator Replacement

**DOI:** 10.36660/abc.20230386i

**Authors:** Lucas Bassoli de Oliveira Alves, Katia Regina Silva, Jacson Venancio Barros, Fernando Antonio Basile Colugnati, Martino Martinelli, Roberto Costa

**Affiliations:** 1 Brazil Instituto do Coração do Hospital das Clínicas da Faculdade de Medicina da Universidade de São Paulo, São Paulo, SP – Brazil; 2 Brazil Faculdade de Medicina da Universidade de São Paulo (FMUSP), São Paulo, SP – Brazil; 3 Brazil Universidade Federal de Juiz de Fora, Juiz de Fora, MG – Brazil

**Keywords:** Artificial Pacemaker, Postoperative Complications, Patient Readmission, Health Evaluation

## Abstract

**Background:**

The use of artificial cardiac pacemakers has grown steadily in line with the aging population.

**Objectives:**

To determine the rates of hospital readmissions and complications after pacemaker implantation or pulse generator replacement and to assess the impact of these events on annual treatment costs from the perspective of the Unified Health System (SUS).

**Methods:**

A prospective registry, with data derived from clinical practice, collected during index hospitalization and during the first 12 months after the surgical procedure. The cost of index hospitalization, the procedure, and clinical follow-up were estimated according to the values reimbursed by SUS and analyzed at the patient level. Generalized linear models were used to study factors associated with the total annual treatment cost, adopting a significance level of 5%.

**Results:**

A total of 1,223 consecutive patients underwent initial implantation (n=634) or pulse generator replacement (n=589). Seventy episodes of complication were observed in 63 patients (5.1%). The incidence of hospital readmissions within one year was 16.4% (95% CI 13.7% - 19.6%) after initial implants and 10.6% (95% CI 8.3% - 13.4%) after generator replacements. Chronic kidney disease, history of stroke, length of hospital stays, need for postoperative intensive care, complications, and hospital readmissions showed a significant impact on the total annual treatment cost.

**Conclusions:**

The results confirm the influence of age, comorbidities, postoperative complications, and hospital readmissions as factors associated with increased total annual treatment cost for patients with pacemakers.

## Introduction

The use of artificial cardiac pacemakers has grown steadily in line with the aging population. This type of treatment has been performed with low rates of perioperative complications and with a proven effect in increasing survival and remission of symptoms.^1-3^ However, studies based on data analysis from administrative systems have demonstrated a progressive increase in the rates of postoperative complications and hospital readmissions, which have been mainly explained by the frailty and comorbidities of this population.^4-8^

Postoperative complications and hospital readmissions are important indicators of care quality and have therefore been increasingly studied.^9-15^ In addition to the negative impacts they cause on patients’ health, these events are one of the main sources of unexpected costs for the health system, resulting in an operational inefficiency of hospital beds and a reduction in the capacity of specialized services.^16-19^

Even so, the main gap in this knowledge area concerns the lack of data resulting from real clinical practice, since most economic evaluation studies on artificial cardiac pacing have been centered on statistical modeling methods based on data from controlled clinical studies.^20,21^ Although these studies have great scientific value, extrapolating the results to our context in Brazil is not always possible, especially because they involve homogeneous population samples with restricted clinical conditions and controlled treatments, which are difficult to reproduce within the specific care model in Brazil.

Thus, the purpose of the present study was to determine the rates of hospital readmissions and complications after pacemaker implantation or pulse generator replacement and to evaluate the impact of these events on the annual costs of treating patients with pacemakers from the perspective of the Unified Health System in Brazil (*Sistema Único de Saúde - SUS*).

## Methods

### Study design and location

This is a prospective registry with data derived from clinical care practice carried out in a tertiary cardiology hospital located in the city of São Paulo, Brazil.

Data were collected at four different moments: at the index hospitalization related to the surgical procedure, and at 30 days, 6 months, and 12 months after hospital discharge (Central Illustration).

### Study population

All adult patients undergoing initial pacemaker implantation or pulse generator replacement were consecutively included, regardless of the clinical indication and the surgical technique used. Patients who had their treatment paid for by private sources of financing or who required procedures associated with pulse generator replacement, such as implantation or removal of leads, were not included.

### Estimation of treatment costs

The method chosen to estimate costs was macro-costing, considering only the direct costs of treatment reimbursed by the *SUS* for our institution.^22^

A repository was built with individualized data from all patients included in the study from the *SUS* billing databases to assess the costs. The Hospital Admission Authorization (*Autorização de Internação Hospitalar - AIH*) was the basic unit for calculating costs related to hospital admission episodes, while the High-Cost Procedure Authorization (*Autorização de Procedimento de Alto Custo - APAC*) and the Outpatient Production Bulletin (*Boletim de Produção Ambulatorial - BPA*) were the systems used to assess costs at an outpatient level.

Hospital services (ward or intensive unit daily rates, hospital materials, imaging, laboratory tests, medications, concomitant therapies) and professional fees for medical services were considered to estimate the total cost of the index hospitalization. These costs were calculated based on fixed amounts that were reimbursed by the *SUS* upon presentation of the *AIH* after the patient’s discharge.

The procedure cost, including the cardiac device (pulse generator and leads), and other supplies, was calculated following the Table of Procedures, Medications, Orthoses, Prostheses and Special Materials (Medical Orthoses and Prostheses) of the *SUS*.^23^

Costs related to the clinical follow-up phase included all outpatient care and procedures, diagnostic tests, laboratory tests, as well as hospital readmissions and surgical interventions.

### Study outcomes

The outcomes studied were hospital readmissions, postoperative complications, and treatment costs. All readmission episodes occurring for any reason in the first year after discharge from the index hospitalization were considered. The readmission incidence was measured at two moments: within 30 days after hospital discharge (early readmission) and at the end of the one-year follow-up (late readmission).

Postoperative complications included: pneumothorax, hemothorax, perforations or injuries to cardiac structures, problems in the pulse generator pocket requiring intervention, local or systemic infection related to the device, endocarditis, upper extremity venous thrombosis ipsilaterally to the device, and lead dysfunction.

Treatment costs were represented by the sum of the values in reais (R$), reimbursed by the *SUS* for expenses related to the index hospitalization, the surgical procedure, clinical follow-up of patients during the first 12 months of treatment, and eventual hospital readmissions that occurred during the study period.

### Data collection and management

Study data were collected in electronic forms developed in the REDCap (Research Electronic Data Capture)^24^ software hosted at our Institution. Specific REDCap functions were used to monitor data quality throughout the study.

### Variables studied and statistical analysis

Demographic variables (age, sex, education, employment status), preoperative variables (type of hospitalization, structural heart disease, regular medications, comorbidities, left ventricular ejection fraction obtained by two-dimensional transthoracic echocardiography), surgical variables (type of procedure, pacemaker indication, type of pacemaker) and the index hospital admission characteristics were considered to analyze the results.

Continuous variables were described as median and interquartile range (IQR) and categorical variables were described as absolute and relative frequencies. Pearson’s chi-squared, Fisher’s exact, and Mann-Whitney tests were used to compare the baseline characteristics of the two groups studied.

The hospital readmission incidence was described in percentage probability and 95% confidence intervals (CI), according to the Kaplan-Meier method. The log-rank test was used to compare estimates between the two groups studied.

The Cox proportional hazards regression method was used to study factors associated with hospital readmissions. Variables with p-values < 0.10 in the univariate analysis were selected for the final multivariate model. The results of the final model are presented in Hazard Ratio (HR) and their respective 95%CI. The final model fit was assessed by calculating the model agreement index (C-index). C-index values equal to or greater than 0.70 were considered satisfactory.

The treatment cost is described according to unadjusted (sample) and adjusted (predicted) mean values followed by the 95%CI, obtained using the bootstrap technique for 5,000 non-parametric resamples. Simple and multivariate generalized linear models (GLM) were implemented to identify factors associated with the total annual cost of treatment using the log-link function and the Gamma distribution to model the total annual cost of treatment. Covariates with a p-value less than 0.10 (in univariate analysis) were included in the final multivariate model. All statistical analyses were performed using the R Studio software program, adopting a significance level of 5% for all hypothesis tests.

### Ethical aspects

The study was conducted from January 2014 to December 2018 and was approved by the Institution’s Research Ethics Committee. As this is an observational study with data derived from care practice and obtained directly from hospital systems (electronic patient records and administrative data systems), the study was exempt from the need to sign an Informed Consent Form.

## Results

### Sample composition

A total of 1,418 patients underwent surgical procedures for the initial implantation or replacement of the pulse generator of conventional cardiac pacemakers during the study period. Of these, 44 patients were under 18 years of age and another 151 patients had their treatment paid for by private financing sources and were therefore considered ineligible for the study. The final sample consisted of 1,223 patients, 634 of whom underwent initial implantation and 589 who underwent pacemaker pulse generator replacement.

### Baseline characteristics

The sample had a higher frequency of women and a median age of 73 years (Q1-Q2: 63–81 years). The proportion of women and the median age were higher among patients in the generator replacement group. The frequency of comorbidities was higher in the pacemaker implantation group, as described in Table 1.

**Table 1:** – Baseline characteristics of patients undergoing initial pacemaker implantation or pulse generator replacement

Baseline characteristics	Total sample N = 1,223	Initial implant N = 634	Generator replacement N = 589	p
Female, n (%)	687 (56.2)	334 (52.7)	353 (59.9)	0.010
**Age (years) median (IQR), n (%)**	73.0 (63 – 81)	72.0 (64 – 80)	74.0 (63 – 82)	< 0.001
< 60	230 (18.8)	112 (17.7)	118 (20.0)	0.030
60 – 69	255 (20.9)	149 (23.5)	106 (18.0)	
70 – 79	392 (32.1)	213 (33.6)	179 (30.4)	
80 – 89	296 (24.2)	137 (21.6)	1859 (27.0)	
≥ 90	50 (4.1)	23 (3.6)	27 (4.6)	
Elementary education, n (%)	864 (70.6)	423 (66.7)	441 (74.8)	0.621
Retired, n (%)	544 (44.5)	267 (42.1)	277 (47.0)	0.087
**Comorbidities, n (%)**				
Hypertension	872 (71.3)	460 (72.6)	412 (69.9)	0.314
Diabetes mellitus	306 (25.0)	180 (28.4)	126 (21.4)	0.005
Valve disease	241 (19.7)	141 (22.2)	100 (17.0)	0.021
Atrial fibrillation	259 (21.2)	125 (19.7)	134 (22.7)	0.208
Coronary artery disease	173 (14.1)	112 (17.7)	61 (10.4)	< 0.001
Chronic kidney disease	110 (9.0)	72 (11.4)	38 (6.4)	0.003
Brain stroke	91 (7.4)	58 (9.1)	33 (5.6)	0.018
**Structural heart disease, n (%)**				
Non-ischemic	181 (14.8)	93 (14.7)	88 (14.9)	0.852
Ischemic	46 (3.7)	27 (4.2)	19 (3.2)	0.355
Chagasic	199 (16.3)	97 (15.3)	102 (17.3)	0.310
LV ejection fraction < 40%, n (%)	72 (5.9)	38 (6.0)	34 (5.7)	0.467
**Pacemaker indication, n (%)**				
Sinus node disease	123 (10.1)	61 (9.6)	62 (10.5)	0.102
Advanced atrioventricular @@block	1.028 (84.0)	527 (83.1)	501 (85.0)	
Other indications	72 (5.9)	46 (7.2)	26 (4.4)	
**Characteristics of the surgical procedure, n (%)**				
Dual chamber pacemaker	1.059 (86.6)	549 (86.6)	510 (86.6)	0.998
Transvenous access	1.209 (98.8)	624 (98.4)	585 (99.3)	0.962
Elective procedure	217 (17.7)	57 (9.0)	160 (27.2)	< 0.001
**Hospitalization, n (%)**				
Emergency hospitalization	531 (43.4)	473 (74.6)	58 (9.8)	< 0.001
Surgery performed on the same day of admission	217 (17.7)	57 (9.0)	160 (27.2)	< 0.001
Hospital stay > 3 days	473 (38.7)	409 (64.5)	64 (10.9)	< 0.001
Post-operative length of stay > 1 day	170 (13.9)	154 (24.3)	16 (2.7)	< 0.001
Post-operative ICU daily rates	101 (8.3)	97 (15.3)	4 (0.7)	< 0.001
**Regular medication use, n (%)**				
Antiplatelet agents	470 (38.4)	243 (38.3)	227 (38.5)	0.924
Oral anticoagulants	139 (11.4)	62 (9.8)	77 (13.1)	0.084
ACEI/ARB	837 (68.4)	394 (62.1)	443 (75.2)	< 0.001
Beta blockers	430 (35.1)	130 (20.5)	300 (50.9)	< 0.001
Diuretics	609 (49.8)	316 (49.8)	293 (49.7)	0.848
Antiarrhythmics	94 (7.7)	43 (6.8)	51 (8.6)	0.243

ARB: angiotensin receptor blocker; ACEI: angiotensin-converting enzyme inhibitor; IQR: interquartile range; ICU: intensive care unit; LV: left ventricle.

Approximately three out of every four initial implants were performed urgently, while a small portion of generator replacement procedures occurred urgently. The rate of surgical procedures performed on the same day of hospital admission was just significantly higher in the generator replacement group. Dual-chamber devices implanted via transvenous access were the most common in the sample. The need for intensive care and postoperative length of stay were significantly higher in the initial implant group (Table 1).

### Deaths, postoperative complications, and hospital readmissions

The median follow-up time was 13.7 months (Q1-Q2: 12.3-14.8 months). Only one patient was lost to follow-up. A total of 109 deaths were observed during the first year of follow-up, representing a cumulative mortality of 8.9% (95%CI 7.4%-10.6%). The causes of death are described in Table 2.

**Table 2:** – Rate of postoperative complications, hospital readmissions, and deaths after initial pacemaker implantation or pulse generator replacement

Outcomes	Total sample N = 1,223	Initial implant N = 634	Generator replacement N = 589
**Postoperative complications, n (%)**			
Pneumothorax	12 (1.0)	11 (1.7)	1 (0.2)
Cardiac tamponade	3 (0.2)	3 (0.5)	0 (-)
Lead displacement	11 (0.9)	10 (1.6)	1 (0.2)
Lead dysfunction	8 (0.7)	1 (0.2)	7 (1.2)
Problems in the connection between the generator and the leads	1 (0.1)	1 (0.2)	0 (-)
Pocket complications	20 (1.6)	8 (1.3)	12 (2.3)
Device infection	8 (0.7)	5 (0.8)	3 (0.5)
Deep vein thrombosis	4 (0.3)	3 (0.5)	1 (0.2)
Muscle stimulation	3 (0.2)	1 (0.7)	2 (0.3)
Any complications	63 (5.2)	38 (6.0)	25 (4.2)
**Early hospital readmissions, n (%)**			
Pacemaker related	13 (1.1)	9 (1.4)	4 (0.7)
Heart failure	5 (0.4)	5 (0.8)	0 (-)
Other cardiovascular causes	1 (0.1)	0 (-)	1 (0.2)
Non-cardiovascular	14 (1.1)	13 (2.2)	1 (0.2)
**Late hospital readmissions, n (%)**			
Pacemaker related	17 (1.4)	5 (0.8)	12 (2.0)
Heart failure	20 (1.6)	14 (2.2)	6 (1.0)
Other cardiovascular causes	28 (2.3)	10 (1.6)	18 (3.0)
Non-cardiovascular	64 (5.2)	45 (7.1)	19 (3.2)
**Deaths, n (%)**			
Pacemaker related	4 (0.3)	3 (0.5)	1 (0.2)
Heart failure	6 (0.5)	2 (0.3)	4 (0.7)
Other cardiovascular causes	29 (2.4)	14 (2.2)	15 (2.5)
Non-cardiovascular	63 (5.1)	46 (7.2)	17 (2.9)
Undetermined cause	7 (0.6)	5 (0.8)	2 (0.3)

In addition, 70 episodes of complications were detected in 63 patients. Among the postoperative complications, 27 occurred during the index hospitalization. These complications significantly increased the length of hospital stay and the need for intensive care. The median length of stay was 5.0 days (Q1-Q2: 2.5-12) in the group that presented complications, and 1.0 days (Q1-Q3: 0-1.0) in the group that did not present complications. The types of postoperative complications are listed in Table 2.

The readmission incidence within 30 days was 4.3% (95%CI: 3.0%-6.2%) for the initial implant group and 1.0% (95%CI: 0.5%- 2.3%) for the generator replacement group. Hospital readmissions at the end of the first year of follow-up occurred in 16.4% (95%CI: 13.7%-19.6%) of patients undergoing initial implantation and in 10.6% (95%CI: 8.3%-13.4%) of patients undergoing generator replacement (Figure 1). The causes of hospital readmissions are described in Table 2 and the independent factors for their occurrence are described in Table 3.

**Figure 1:** – Probability of readmission within 12 months according to the type of surgical procedure performed.

**Table 3:** – Predictors of hospital readmission after initial pacemaker implantation or pulse generator replacement

Risk factors	Hazard Ratio (95%CI)	p
**Pacemaker implant ^**1**^**		
Age ≥ 90 years	1.29 (0.59 – 2.82)	0.522
Diabetes mellitus	1.47 (0.94 – 2.29)	0.087
Chronic kidney disease	2.02 (1.21 – 3.39)	0.007
Atrial fibrillation	1.72 (0.99 – 2.96)	0.051
Previous stroke	1.20 (0.64 – 2.21)	0.566
Structural heart disease	1.56 (1.01 – 2.40)	0.043
Indication for pacemaker implantation		
Atrioventricular block	reference	-
Sinus node disease	0.75 (0.34 – 1.66)	0.482
Other indications	1.24 (0.60 – 2.54)	0.563
Regular use of oral anticoagulants	0.70 (0.36 – 1.38)	0.309
Single chamber pacemaker	2.61 (1.55 – 4.41)	< 0.001
Postoperative hospital stay > 1 day	1.07 (0.63 – 1.81)	0.799
Hospital stay > 3 days	0.90 (0.55 – 1.49)	0.692
Need for ICU in the postoperative period	1.14 (0.66 – 1.97)	0.640
Pacemaker-related complications	5.94 (3.33 – 10.58)	< 0.001
**Pacemaker pulse generator replacement ^**2**^**		
Age ≥ 80 years	2.52 (1.48 – 4.29)	< 0.001
Male	1.41 (0.82 – 2.42)	0.214
Chronic kidney disease	2.17 (0.99 – 4.74)	0.052
Atrial fibrillation	1.18 (0.58 – 2.39)	0.640
Previous stroke	2.93 (1.36 – 6.28)	0.006
Regular use of oral anticoagulants	1.03 (0.45 – 2.37)	0.935
Hospital stay > 3 days	1.67 (0.78 – 3.56)	0.181
Pacemaker-related complications	25.65 (12.70 – 51.60)	< 0.001

^1^ n = 596; C-index = 0.757. ^2^ n = 582; C-index = 0.815.

### Cost of treatment in the first year after the procedure

The *SUS* reimbursed our institution with approximately R$10.6 million for the treatment of patients included in the study. The cardiac device, including the leads and pulse generator, was the main component for these costs and represented more than 70 % of the total annual expenditure. Table 4 presents a detailed description of the costs attributed to treating patients in the initial implant and pulse generator replacement groups.

**Table 4:** – Description of expenses attributed to the index hospitalization, clinical follow-up, and the total amount for the treatment of patients with pacemakers

Expense components	Mean	95%CI	Total amount	Total
**Pacemaker implant**				
**Annual total**	R$ 10,172	(9,770 – 10,620)	R$ 6,449,363	100%
**Device implant**				
**Total**	R$ 8,934	(8,702 – 9,205)	R$ 5,664,163	87.8%
Device (MOP)	R$ 7,162	(7,110 – 7,216)	R$ 4,540,877	70.4%
Hospitalization	R$ 1,224	(1,155 – 1,307)	R$ 776,400	12.0%
Intensive Care Unit	R$ 547	(366 – 750)	R$ 346,886	5.4%
**Outpatient follow-up**				
Consultations or Procedures	R$ 743	(586 – 945)	R$ 471,157	7.3%
**Hospital readmissions**				
Total	R$ 495	(276 – 754)	R$ 314,043	4.9%
Device (MOP)	R$ 53	(19 – 97)	R$ 33,980	0.5%
Hospitalization	R$ 442	(242 – 680)	R$ 280,063	4.4%
**Pulse generator replacement**				
**Annual total**	R$ 7,092	(6,750 – 7,514)	R$ 4,177,440	100%
**Generator replacement**				
Total	R$ 6,029	(5,994 – 6,068)	R$ 3,551,176	85.0%
Device (MOP)	R$ 5,125	(5,100 – 5,150)	R$ 3,018,836	72.3%
Hospitalization	R$ 886	(871 – 904)	R$ 522,168	12.5%
Intensive Care Unit	R$ 17	(0,86 – 42,3)	R$ 10,172	0.2%
**Outpatient follow-up**				
Consultations or Procedures	R$ 510	(469 – 555)	R$ 300,350	7.2%
**Hospital readmissions**				
Total	R$ 553	(235 – 956)	R$ 325,914	7.8%
Device (MOP)	R$ 300	(77 – 593)	R$ 176,908	4.2%
Hospitalization	R$ 253	(98 – 465)	R$ 149,007	3.6%

MOP: Medical Orthosis and Prosthetic.

The generalized linear model demonstrated that age, chronic kidney disease, previous stroke, hospital stay longer than one day, need for postoperative intensive care, complications, and hospital readmissions were significantly associated with total annual treatment costs. Age was the only variable that was inversely related to the total cost of treatment, regardless of the procedure performed (Table 5).

**Table 5:** – Multivariate model of factors associated with the annual cost of treating patients with cardiac pacemakers

Risk factors	Exp B (95%IC)	p
**Age groups (years)**		
< 60	Reference	--
60 – 69	0.97 (0.93 – 1.01)	0.149
70 – 79	0.96 (0.92 – 1.00)	0.035
80 – 89	0.92 (0.89 – 0.96)	< 0.001
≥ 90	0.87 (0.81 – 0.94)	< 0.001
Diabetes mellitus	1.03 (1.00 – 1.06)	0.086
Valve disease	1.00 (0.96 – 1.03)	0.820
Atrial fibrillation	1.04 (1.00 – 1.08)	0.076
Coronary artery disease	1.01 (0.97 – 1.05)	0.579
Chronic kidney disease	1.07 (1.02 – 1.12)	0.009
Previous stroke	1.08 (1.02 – 1.13)	0.005
Structural heart disease	1.00 (0.97 – 1.03)	0.899
Regular use of oral anticoagulants	0.96 (0.91 – 1.01)	0.097
Emergency hospitalization	1.00 (0.96 – 1.04)	0.990
Procedure carried out on an elective basis	0.99 (0.96 – 1.03)	0.787
Hospital stay > 3 days	1.00 (0.98 – 1.07)	0.298
Post-operative hospital stay > 1 day	1.00 (0.98 – 1.07)	0.298
Postoperative intensive care unit daily rates	1.39 (1.31 – 1.47)	< 0.001
Pacemaker-related complication	1.17 (1.09 – 1.25)	< 0.001
Hospital readmission	1.56 (1.48 – 1.66)	< 0.001

Exp B: beta coefficient exponent.

Hospital readmission during clinical follow-up and the need for postoperative intensive care during the index hospitalization were the main factors associated with the increase in the treatment cost, as detailed in Figure 2.

**Figure 2:** – Factors associated with the average increase in the total annual cost of treating patients with a pacemaker.

## Discussion

This prospective study with real-world data showed that postoperative complications and hospital readmissions are frequent after the initial implant, as well as after pacemaker pulse generator replacement. Regardless of the reason for these readmissions, the economic impact on the total cost of healthcare for the public healthcare system was significant.

Although patients undergoing initial implantation or pulse generator replacement are part of the same patient population, significant differences were observed in the clinical and demographic profile of these two subgroups, with a higher proportion of women, lower prevalence of comorbidities, and a higher frequency of cardiovascular medication use in those undergoing pulse generator replacement. Furthermore, most pulse generator replacements were performed in elective admissions, while initial implants were mostly performed on an emergency basis. These differences influenced the outcome of the procedures, resulting in longer hospital stays, the need for intensive care unit admission, and hospital readmissions in patients undergoing initial implants. Although current pulse generators have a useful life expectancy of approximately 10 years, the median age of patients at the pulse generator replacement time exceeded the age of the initial implant group by only two years. This finding can be explained by the high rate of patients who do not undergo pulse generator replacement due to their advanced age at the initial implant time.

The rates of postoperative complications and early hospital readmissions related to the surgical procedure or cardiac device found in this study were lower than those reported in studies based on large US administrative databases, in which the 30-day hospital readmission rate ranged from 8.5% to 11.3%.^9,10^ In the present study, pneumothorax or cardiac tamponade (2.2%) and lead-related complications (1.9%) were more frequent in the initial implant group, and their rates were similar to those reported in the FOLLOWPACE study (2.7% and 3.3%, respectively).^11^

Despite the lower total readmission rate in the generator replacement group, the frequency of procedure-related readmissions was higher in this subgroup, especially after the first 30 days of follow-up. These readmissions were related to complications in the pulse generator pocket, lead dysfunctions, and device-related infection. Similar to what has been reported in other studies, these complications generally occurred late, requiring readmission and surgical revision.^12-19^

The one-year readmission rate was 16.4% after initial implants and 10.6% in the generator replacement group. Age, chronic kidney disease, underlying heart disease, single-chamber pacemaker, and postoperative complications significantly increased the risk of readmissions, in agreement with other publications.^9,10,14^ Likewise, chronic kidney disease, previous stroke, postoperative complications, and readmissions were associated with higher healthcare costs in the first year, both after initial implantation and after pulse generator replacement. Monitoring and knowledge of complication rates by the medical team, continuous training of teams to identify patients at higher risk, in addition to multidisciplinary follow-up of these patients are potentially accessible measures that offer a good opportunity to improve complication rates and readmissions after the procedure.

Our analysis revealed a significant economic impact of the device cost on the total treatment cost (around 70%). Other publications have already demonstrated the disproportion between the cost of the cardiac device concerning other expenses, such as hospitalization fees, laboratory tests, medications, and professional fees.^18,19^

This study presents some limitations that must be considered when interpreting the results. Although a very representative sample was included, this analysis reflects the care practices of a public tertiary cardiology center, which is also a training center for specialists in artificial cardiac pacing. Direct costs were calculated based on fixed values, or packages that were reimbursed by the *SUS*, and it was not possible to conduct a micro-costing analysis with detailed data on each resource used and the unit costs corresponding to these resources. Finally, long-term follow-up of this population is especially important to provide more robust evidence on the potential impact of late complications on healthcare costs, which are often underreported in this setting.

## Conclusion

Follow-up for up to one year of patients undergoing initial cardiac pacemaker implantation or a pulse generator replacement procedure allowed us to determine the occurrence rate of postoperative complications and hospital readmissions, identify risk factors for these events, and verify that these events resulted in a significant increase in the treatment cost. The findings of the present study suggest that identifying patients who are at greater risk of experiencing these events and implementing specific care routines for their outpatient follow-up can result in a significant reduction in spending on these types of procedures.
